# Cardiac output in idiopathic normal pressure hydrocephalus: association with arterial blood pressure and intracranial pressure wave amplitudes and outcome of shunt surgery

**DOI:** 10.1186/2045-8118-8-11

**Published:** 2011-02-04

**Authors:** Per K Eide

**Affiliations:** 1 Department of Neurosurgery, Oslo University Hospital - Rikshospitalet, 0027 Oslo, Norway; 2 Faculty of Medicine, University of Oslo, Oslo, Norway

## Abstract

**Background:**

In patients with idiopathic normal pressure hydrocephalus (iNPH) responding to shunt surgery, we have consistently found elevated intracranial pressure (ICP) wave amplitudes during diagnostic ICP monitoring prior to surgery. It remains unknown why ICP wave amplitudes are increased in these patients. Since iNPH is accompanied by a high incidence of vascular co-morbidity, a possible explanation is that there is reduced vascular compliance accompanied by elevated arterial blood pressure (ABP) wave amplitudes and even altered cardiac output (CO). To investigate this possibility, the present study was undertaken to continuously monitor CO to determine if it is correlated to ABP and ICP wave amplitudes and the outcome of shunting in iNPH patients. It was specifically addressed whether the increased ICP wave amplitudes seen in iNPH shunt responders were accompanied by elevated CO and/or ABP wave amplitude levels.

**Methods:**

Prospective iNPH patients (29) were clinically graded using an NPH grading scale. Continuous overnight minimally-invasive monitoring of CO and ABP was done simultaneously with ICP monitoring; the CO, ABP, and ICP parameters were parsed into 6-second time windows. Patients were assessed for shunt surgery on clinical grade, Evan's index, and ICP wave amplitude. Follow-up clinical grading was performed 12 months after surgery.

**Results:**

ICP wave amplitudes but not CO or ABP wave amplitude, showed good correlation with the response to shunt treatment. The patients with high ICP wave amplitude did not have accompanying high levels of CO or ABP wave amplitude. Correlation analysis between CO and ICP wave amplitudes in individual patients showed different profiles [significantly positive in 10 (35%) and significantly negative in 16 (55%) of 29 recordings]. This depended on whether there was also a correlation between ABP and ICP wave amplitudes and on the average level of ICP wave amplitude.

**Conclusions:**

These results gave no evidence that the increased levels of ICP wave amplitudes seen in iNPH shunt responders prior to surgery were accompanied by elevated levels of ABP wave amplitudes or elevated CO. In the individual patients the correlation between CO and ICP wave amplitude was partly related to an association between ABP and ICP wave amplitudes which can be indicative of the state of cerebrovascular pressure regulation, and partly related to the ICP wave amplitude which can be indicative of the intracranial compliance.

## Background

The clinical condition idiopathic normal pressure hydrocephalus (iNPH) consists of unsteady gait, urinary incontinence, dementia, and enlarged lateral ventricles [1]. The preferred treatment is insertion of a shunt for drainage of cerebrospinal fluid (CSF), which gives good clinical results and a favourable complication profile [2,3]. Despite recent advances in the treatment of iNPH, the rationale for treatment is based on a limited understanding of its pathophysiology. Possible important pathophysiological mechanisms include reduced regional/global cerebral blood flow (CBF) indicative of chronic ischemia [4-8], impaired intracranial compliance (ICC) [9-12], and impaired cerebral pulsation absorber capacity causing cerebral capillary damage [13,14]. We have considered impaired ICC to be a crucial pathophysiological mechanism behind iNPH [11,12], and have previously reported that the ICC becomes reduced when the amplitude of intracranial pressure (ICP) waves increases [15].

In a previous study of 130 shunted iNPH patients, we had significant clinical improvement in response to shunt treatment in 9 out of 10 patients with high ICP wave amplitudes (MWA_ICP_; ≥4 mmHg on average and ≥5 mmHg in 10% of recording time), but in only 1 of 10 patients with low ICP wave amplitudes [11]. Given these promising results, it is important to understand better the causes for elevated ICP wave amplitudes in iNPH. In general, the ICP wave is determined by many factors such as changes in cerebral blood volume (CBV) during the cardiac cycle [16-18], and possibly by alterations in cardiac output (CO) [19,20]. In iNPH, vascular factors might be particularly important since this condition is accompanied with a high incidence of vascular co-morbidity such as hypertension, ischemic heart disease and diabetes [5,21-26]. Obviously, vascular disease might affect how the ABP waves that are created by the cardiac contractions are transferred into the ICP waves. From previous experimental work, it has been established that during impaired cerebrovascular pressure regulation, the ABP waves are transferred more passively to the ICP waves [27-29]. Accordingly, we hypothesised that in iNPH patients with vascular disease, increased ICP wave amplitudes might be a reflection of increased ABP wave amplitudes and possibly increased CO.

The present study was undertaken to investigate whether CO is related to ABP and ICP wave amplitudes and to shunt response in iNPH patients, in particular, whether the increased ICP wave amplitudes seen in iNPH patients that subsequently respond to shunt surgery are accompanied by increased levels of CO or ABP wave amplitudes.

## Methods

### Patients

The study included 29 consecutive patients evaluated for clinical iNPH at the Department of Neurosurgery, Oslo University Hospital - Rikshospitalet, as part of our standardized pre-operative evaluation of iNPH patients. One additional patient was excluded from the study due to technical problems with the ABP signal. The study was approved by the Regional Ethical Committee of Health Region South-East in Norway (S-07362b) and by Rikshospitalet (07/5870). Written informed consent was obtained from the patients before the study inclusion.

### Diagnostic work-up and follow-up after shunting

The patients were referred with suspected iNPH from local neurological departments based on their clinical symptoms and findings (gait disturbance, incontinence, and dementia) combined with radiological ventriculomegaly. Based on our findings at neurological examination, the severity of clinical iNPH was graded using a NPH grading scale ranging from a best score of 15 to a worst score of 3 (see additional file 1, Table showing NPH grading scale) which assesses the combined severity of gait disturbance, urinary incontinence and dementia [11]. The criteria for surgical treatment were based on symptoms (NPH grading), increased ventricular size (Evan's index >0.3), and findings from the diagnostic ICP monitoring. The surgical treatment was insertion of a ventricular-peritoneal (VP) shunt with a HAKIM™ programmable valve shunt system (Codman & Shurtleff, Inc. Le Locle, Switzerland), with an opening pressure at implantation of 12 cm H_2_O.

Follow-up examination (NPH grading) was performed in our out-patient clinic 12 months after surgery. We defined an increase ≥2 in NPH score at 12 months as representative of clinical improvement; the surgically treated patients were categorized either as responders (change in NPH score ≥2) or non-responders (change in NPH score <2), respectively.

### Monitoring and analysis of CO, ABP and ICP wave amplitudes

The design of the study was to simultaneously monitor CO, ABP, and ICP overnight using a computerized recording system, as part of the diagnostic procedure (above). The patients were kept supine in bed during the entire recording.

Continuous monitoring of CO was performed with the LiDCO™*plus *(software version 4.0, LiDCO Ltd., Cambridge, UK). This is a minimally invasive technique incorporating two methods: a continuous arterial waveform analysis system (PulseCO), coupled to a single-point lithium indicator dilution calibration system (LiDCO) [30,31]. As previously described [31], the calibration procedure involves injection of 0.3 mmol lithium chloride through a peripheral line. The lithium is detected by an external lithium ion-sensitive external electrode connected to the peripheral arterial line, which enables monitoring of the actual CO. In all the present patients, monitoring of hemodynamic parameters was done after calibration. The primary measure for this study was the CO, but the software also computed the stroke volume (SV), heart rate (HR), oxygen consumption (DO_2_), and systemic vascular resistance (SVR). ABP was measured continuously in the right radial artery using the Truwave PX-600F Pressure Monitoring Set (Edwards Life sciences LLC, Irvine, CA, USA). The ABP sensor was placed at the level of the heart. ICP was monitored continuously using a solid sensor (Codman MicroSensor™, Johnson & Johnson, Raynham, MA, USA), introduced 1-2 cm into the frontal brain parenchyma through a small burr hole and a minimal opening in the dura, as previously described [11].

**Figure 1:**
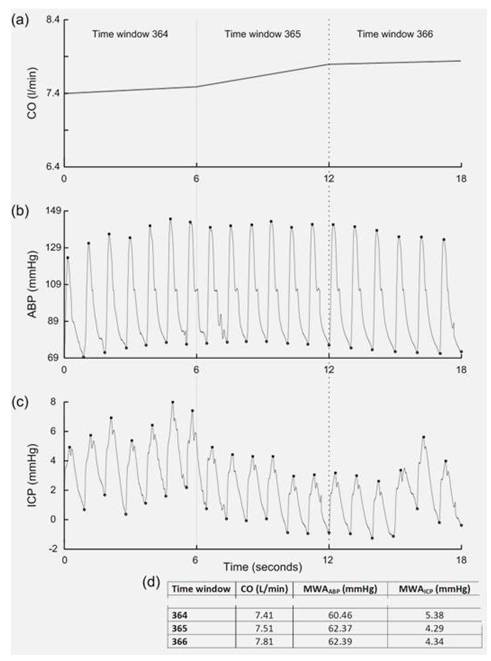
**Illustration of continuous monitoring in patient #5**. Recordings of three consecutive 6-s time windows (time windows 364-366) from a patient, showing (a) the averaged cardiac output (CO) over each 6-s time window, (b) the arterial blood pressure (ABP) signal, and (c) the intracranial pressure (ICP) signal for the same time period. For each of the 6-s time windows the calculated CO, mean ABP wave amplitude (MWA_ABP_), and mean ICP wave amplitude (MWA_ICP_) is presented in (d).

The continuous ABP and ICP waveforms were sampled at 200 Hz, digitized using the Sensometrics^® ^Pressure Logger (dPCom AS, Oslo, Norway) and analyzed using Sensometrics^® ^software (dPCom AS). The LiDCO™plus and Sensometrics^® ^software's had identical time reference. An example of the simultaneous recordings is illustrated in Figure 1. Cardiac output retrieved from the LiDCO™plus Hemodynamic Monitor was averaged over 6-s time windows (Figure 1a). As previously described [32], the single ABP waves (Figure 1b) and ICP waves (Figure 1c) were automatically identified, each wave being characterized by the amplitude, rise time, and rise time coefficient. For each consecutive 6-s time window, cardiac output, mean ABP wave amplitude and mean ICP wave amplitude were computed, and exported to a spread sheet program for further analysis (Figure 1d).

During the pre-operative work-up, the patients were divided into two management groups: group A with elevated ICP wave amplitudes (MWA_ICP_) and group B with low ICP wave amplitudes (Table 1), according to our previously-established criteria for increased ICP wave amplitudes during diagnostic ICP monitoring [11]. Thus *"elevated" *ICP wave amplitudes were defined as: average of mean ICP wave amplitude ≥4 mmHg, combined with mean ICP wave amplitude ≥5 mmHg in ≥ 10% of the time recording during the period 23.00h - 07.00h [11]. Conversely, *"low" *ICP wave amplitudes were defined as: average of mean ICP wave amplitude <4 mmHg, combined with mean ICP wave amplitude ≥5 mmHg in <10% of the time recording during the same period [11].

**Table 1:** Demographic, clinical, radiological and management data of the two management groups

	**Management Groups**		
		
	**Group A (elevated MWA**_**ICP**_**)**	**Group B (low MWA**_**ICP**_**)**	**Significance**
			
***Demographic***			
Patients (n)	20 (69%)	9 (31%)	
Age (yrs)	76 (53 - 84)	78 (66 - 80)	ns
Gender (F/M)	12/8	3/6	ns
BMI	24.3 (15.1 - 29.4)	24.0 (20.0 - 31.3)	ns
			
***Vascular co-morbidity***			
Diabetes	5 (25%)	1 (11%)	ns
Hypertension	3 (15%)	3 (33%)	ns
Cardiovascular	8 (40%)	3 (33%)	ns
Cerebrovascular	5 (25%)	2 (22%)	ns
Cardiac arrhythmia	3 (15%)	3 (33%)	ns
			
***Radiology***			
Evan's index	0.37 (0.30 - 0.44)	0.38 (0.33 - 0.42)	ns
			
***Symptoms***			
Duration of symptoms (yrs)	2.0 (0.4 - 10)	2.0 (0.8 - 6.0)	ns
Preoperative NPH score	10 (7 - 13)	10 (9 - 11)	ns
			
***Treatment***			
Conservative (n)	1	6	
Shunt (n)	19	3	
			
***Outcome***			
Shunt Non-responders	3 (16%)	3 (100%)	
Shunt Responders	16 (84%)	-	

Management groups were defined according to published criteria [11]. BMI: Body mass index. Significance: ns = non-significant; Fischer Exact test or one-way ANOVA).

The primary comparison was to determine differences in cardiac-, and ABP/ICP-derived parameters between management groups A and B (Table 2). Secondarily, for each patient recording the following Pearson correlation coefficients were determined: (i) between CO and ABP wave amplitude (CO/MWA_ABP _correlation); (ii) between CO vs. ICP wave amplitude (CO/MWA_ICP _correlation); and (iii) between ABP and ICP wave amplitude (MWA_ABP_/MWA_ICP _correlation; Table 3).

**Table 2:** Cardiac and pressure data for the two management groups

	**Management groups**		
		
	**Group A (elevated MWA**_**ICP**_**)**	**Group B (low MWA**_**ICP**_**)**	**Significance**
			
***Duration of recording***			
Accepted 6-s time windows	7,329 (1,022 - 12,388)	7,416 (3,370 - 9,143)	ns
			
***Cardiac parameters***			
CO (l/min)	5.6 (3.6 - 9.6)	5.5 (3.7 - 6.4)	ns
SV (ml/beat)	81.1 (48.4 - 134.5)	82.1 (52.9 - 103.1)	ns
HR (beats/min)	68.6 (58.8 - 89.7)	67.1 (62.3 - 87.9)	ns
DO_2 _(ml ∙ min ∙ m^2^)l)	1,006 (593 - 2,104)	923 (759 - 1,097)	ns
SVR (dyne ∙ s∙ cm^5^)	1,448 (508 - 2,157)	1,426 (997 - 2,144)	ns
			
***ABP parameters***			
Mean ABP (mmHg)	90.7 (72.5 - 112.3)	96.2 (78.0 - 108.1)	ns
Mean ABP wave amplitude (MWA_ABP_; mmHg)	68.6 (52.0 - 110.0)	60.9 (42.1 - 77.6)	ns
			
***ICP parameters***			
Mean ICP (mmHg)	6.4 (2.3 - 11.9)	4.7 (2.1 - 10.8)	ns
Mean ICP wave amplitude (MWA_ICP_; mmHg)	5.6 (4.0 - 10.2)	3.4 (2.8 - 4.0)	c
Percentage mean ICP wave amplitude (MWA_ICP_) ≥5 mmHg	68 (11 - 100)	2 (1 - 7)	c
			
***CPP***			
Mean CPP (mmHg)	87.2 (62.7 - 112.1)	92.9 (75.5 - 107.7)	ns
			
***Correlation values***			
CO/MWA_ABP_	0.37 (0.02 - 0.80)	0.55 (0.09 - 0.87)	ns
CO/MWA_ICP_	-0.06 (-0.24 - 0.21)	0.07 (-0.12 - 0.39)	a
MWA_ABP_/MWA_ICP_	0.05 (-0.29 - 0.32)	0.09 (-0.15 - 0.35)	ns

Management groups were defined according to published criteria [11]. Significance: ^a^*P *< 0.05; ^c^*P *< 0.001 (one-way ANOVA).

**Table 3:** Pearson product-moment correlation coefficients between different measured variables

Patient number	Number of 6s recordings	**CO/MWA**_**ABP**_	**CO/MWA**_**ICP**_	**MWA**_**ABP**_**/MWA**_**ICP**_	Management group	Outcome Category
1	3,370	0.55^**c**^	-0.11^c^	0.09^**c**^	B	C
2	12,388	0.54^**c**^	-0.12^c^	-0.22^**c**^	A	NR
3	4,776	0.08^**c**^	0.08^c^	0.32^**c**^	A	C
4	10,627	0.31^**c**^	0.09^c^	0.01	A	R
5	6,562	0.66^**c**^	-0.18^c^	-0.22^**c**^	A	R
6	7,627	0.52^**c**^	-0.24^c^	-0.29^**c**^	A	R
7	6,081	0.25^**c**^	-0.18^c^	-0.14^**c**^	A	R
8	8,501	0.87^**c**^	0.23^c^	0.23^**c**^	B	NR
9	8,172	0.85^**c**^	0.37^c^	0.35^**c**^	B	NR
10	1,022	0.25^**c**^	-0.003	0.14^**c**^	A	R
11	10,715	0.63^**c**^	0.18^c^	0.26^**c**^	A	R
12	4,519	0.60^**c**^	-0.08^c^	0.003	B	C
13	6,089	0.80^**c**^	-0.04^a^	0.04^**a**^	A	R
14	8,518	0.12^**c**^	-0.19^c^	0.23^**c**^	A	NR
15	8,202	0.59^**c**^	-0.03^a^	0.05^**c**^	A	R
16	7,030	0.14^**c**^	-0.05^c^	0.21^**c**^	A	R
17	3,538	0.25^**c**^	-0.12^c^	-0.15^**c**^	B	C
18	8,239	0.58^**c**^	-0.07^c^	-0.07^**c**^	A	R
19	7,416	0.44^**c**^	0.005	0.18^**c**^	B	C
20	8,117	0.04^**c**^	-0. 08^c^	0.21^**c**^	A	R
21	5,476	0.02	-0.17^c^	0.18^**c**^	A	R
22	8,991	0.09^**c**^	0.39^c^	0.07^**c**^	B	C
23	6,441	0.42^**c**^	-0.12^c^	-0.07^**c**^	A	R
24	9,478	0.15^**c**^	-0.02	0.04^**c**^	A	R
25	9,143	0.29^**c**^	0.07^c^	0.24^**c**^	B	NR
26	5,291	0.12^**c**^	-0.13^c^	-0.03^**a**^	A	NR
27	6,409	0.73^**c**^	0.21^c^	0.23^**c**^	A	R
28	6,467	0.61^**c**^	0.11^c^	0.06^**c**^	B	C
29	8,311	0.57^**c**^	0.15^c^	0.10^**c**^	A	R

***Median***	7,416	0.44	-0.04	0.07		
***Ranges***	1,022 - 12,388	0.02 - 0.87	-0.24 - 0.39	-0.29 - 0.35		

CO: mean cardiac output; MWA_ABP_: mean arterial blood pressure wave amplitude; MWA_ICP_: mean intracranial pressure wave amplitude. Significance: ^a^*P *< 0.05; ^b^*P *< 0.01; ^c^*P *< 0.001 (Pearson correlation coefficient for each individual patient recording). C: Conservative management; NR: Surgical Non-Responder; R: Responder.

The MWA_ABP_/MWA_ICP _correlation has been previously introduced as a tentative indicator of the state of cerebrovascular pressure regulation [33,34]. In this study, the overall MWA_ABP_/MWA_ICP _correlation for each recording was computed, while in previous studies the moving MWA_ABP_/MWA_ICP _correlation has been used [33,34].

### Statistical analysis

Statistical analyses were performed in SPSS, version 12.0 (SPSS Inc., Chicago, IL, USA). Differences between management groups of continuous data were determined by one-way ANOVA. Significant correlations between parameters of individual recordings were determined using the Pearson correlation. Significance was accepted at the 0.05 level.

## Results

### Patients

Of the 29 patients in the study, 20 were in management group A, and 9 in management group B. Shunting was performed on 22 patients, 19/20 from group A and 3/9 from group B. Demographic, clinical, radiological and management data of the two groups are shown in Table 1 and no significant differences were seen between groups.

The continuous monitoring of cardiac and pressure data gave no complications or side-effects. Comparison of the cardiac and arterial pressure data for groups A and B revealed no significant differences between groups in levels of CO or ABP wave amplitude (or other cardiac/ABP parameters; Table 2). However, table 2 shows that the CO/MWA_ICP _correlation was significantly more negative in group A than B. When considering all 29 recordings, there was no significant overall correlation between average values of CO and ICP wave amplitude (Pearson correlation coefficient 0.07, ns; data not shown).

### Shunt response versus cardiac and pressure data

**Figure 2:**
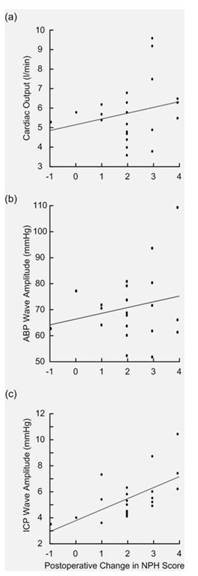
**Cardiac output (CO) arterial blood pressure (ABP) and intracranial pressure (ICP) wave amplitudes plotted against post-shunt changes in NPH score**. The association between change in NPH score 12 months after shunting and the pre-operative overnight average measurements of (a) CO (Pearson correlation coefficient 0.24; ns), (b) ABP wave amplitude (Pearson correlation coefficient 0.21; ns), and (c) ICP wave amplitude (Pearson correlation coefficient 0.61; *P *= 0.003) are presented for the 22 shunted iNPH patients.

The clinical improvement 12 months after shunting (that is, change in NPH score) related poorly to the CO (Figure 2a; ns) and to ABP wave amplitude (Figure 2b; ns), but it correlated highly significantly to the ICP wave amplitude (Figure 2c; *P *= 0.003). Thus, with higher pre-operative ICP wave amplitude levels, better clinical improvement of iNPH symptoms was observed 12 months after shunting (Figure 2c).

While shunt response was related to the level of ICP wave amplitudes, the response to shunting was less dependent on the CO/MWA_ICP _and MWA_ABP_/MWA_ICP _correlation levels (Table 3). In particular, it should be noted that clinical response to shunting was seen in patients with high MWA_ICP _levels (group A) even in the presence of significant positive CO/MWA_ICP _correlation or significant positive MWA_ABP_/MWA_ICP _correlation. Among the 7 shunted patients with significant positive CO/MWA_ICP _correlation, all the 4 shunt responders had elevated MWA_ICP _levels (group A) while the 3 shunt non-responders had low MWA_ICP _levels (group B). Moreover, among the 14 shunted patients with significant positive MWA_ABP_/MWA_ICP _correlation, all the 10 shunt responders had elevated MWA_ICP _levels (group A); while 3 of 4 shunt non-responders had low MWA_ICP _levels (group B).

### The association between CO and ICP wave amplitudes

While a significant positive CO/MWA_ABP _correlation was seen in 28 of 29 patients (97%), significant positive CO/MWA_ICP _correlation was seen in only 10 of 29 patients (35%; Table 3). Most importantly, the patient recordings with significant positive CO/MWA_ICP _correlation also presented with significant positive MWA_ABP_/MWA_ICP _correlation (Table 4). When comparing the data in Table 3 with significant MWA_ABP_/MWA_ICP _correlations, the CO/MWA_ICP _correlation values were significantly more positive in the recordings with positive than negative MWA_ABP_/MWA_ICP _correlation (respective median values 0.07 versus -0.13; *P *= 0.001; ANOVA).

**Table 4:** Distribution of recordings with both significant CO/MWA_ICP _correlation and significant MWA_ABP_/MWA_ICP _correlation

	**MWA**_**ABP**_**/MWA_ICP _correlation**		
		
**CO/MWA_ICP _correlation**	**Significant positive**	**Significant negative**	**Significance**^**a**^
			
Significant positive	9	0	
Significant negative	7	8	*P *= 0.009

^a^Significance of numbers within the matrix was assessed using Fisher's Exact test.

Moreover, as shown in Table 3, significant negative CO/MWA_ICP _correlation (that is, CO declines when ICP wave amplitude increases) was seen in 16 patients, 13 (81%) in group A and 3 (19%) in group B.

## Discussion

This study for the first time reports an association between CO and intracranial pressure dynamics in hydrocephalus patients. One major observation was that the increased ICP wave amplitude levels seen in 20 of the 29 patients were not accompanied with increased levels of CO or ABP wave amplitude (Table 2).

### Patient material

Although there were only 29 patients, the data analysis for each patient was based on a large number of observations; for each patient, recordings between 1,022 and 12,388 6-s time windows of good quality were available for analysis (median 7,416 6-s time windows; Table 3). The computerized recordings allowed comparisons of CO, ABP and ICP wave amplitudes for concurrent 6-s time windows (Figure 1). The methods for physiological monitoring used in this study have previously been described, including methods for ABP and ICP monitoring [32], and the minimal invasive approach for CO monitoring [30]. The PulseCO hemodynamic monitor has been validated against the pulmonary artery catheter method [35], and in various patient groups [36-41].

This cohort of 29 iNPH patients compared well with our recently reported series of 130 surgically-treated iNPH patients regarding age, gender, clinical severity, and a rather high presence of vascular co-morbidity [11]. As in the published series [11], the clinical response to shunting was significantly correlated with increasing levels of ICP wave amplitudes (Figure 2c). In this department, demonstration of increased levels of ICP wave amplitude during diagnostic overnight ICP monitoring is used as an aid for management.

#### The ICP wave amplitude as an indicator of intracranial compliance

During the last few years several studies have focused on a possible important role of impaired ICC for the development of communicating hydrocephalus [9-12]. Both experimental [42] and clinical work [15] indicate that the ICP wave amplitude serves as an indicator of ICC (i.e. the pressure-volume reserve capacity). Recent data using magnetic resonance imaging (MRI) provide evidence that the ICC capacity mostly depends on the contribution of the intracranial compartment rather than the intrathecal [43]. We have interpreted the reduction in ICP wave amplitudes following CSF diversion by extended lumbar drainage [8] or shunt implantation [44] as indicative of improved ICC. Others have used the lumbar infusion test to predict how much the ICP wave amplitudes are reduced following CSF diversion [45].

It is assumed that the ICP wave amplitude represents the pressure response to the intracranial volume change during the cardiac cycle; the net intracranial volume change during the cardiac cycle is about 1 ml [46]. The ICP wave depends on variations in CBV [16,17,47]. Therefore, one criticism against considering the ICP wave amplitude as an indicator of ICC has been that the CBV is an unknown variable, and probably subject to intra- and inter-individual variation. On the other hand, it can be questioned how large an impact such variations in CBV might have on the ICP waveform, given that the net volume change is rather small, and probably ranging between 0.7 and 1.2 ml. Thus, more recently the Cambridge group reported that the ICP wave amplitude of NPH patients was less dependent on alterations in arterial CBV [18].

In the present cohort, there were no differences between the management groups concerning the cardiac (CO, SV, HR and SVR), ABP (mean ABP, mean ABP wave amplitude) or CPP (mean CPP) parameters (Table 2). This study confirms previous observations [34] that there is a non-significant tendency towards higher ABP wave amplitudes in iNPH shunt responders while mean ABP and mean CPP are similar in both the responder/non-responder groups. This study extends previous knowledge by showing that at the group level cardiac measures were close to identical between patients with high or low ICP wave amplitudes. However, although the primary comparison showed no differences in absolute levels of cardiac and blood pressure parameters between groups, the secondary correlation analysis revealed an interesting observation.

#### The MWA_ABP_/MWA_ICP _correlation as an indicator of cerebrovascular pressure regulation

How the ABP waves transfer into the ICP waves depends on the state of cerebrovascular pressure regulation. Previous work on the transfer of ABP waves into ICP waves gave evidence that loss of vasomotor tone of precapillary vessels (disrupted cerebral pressure auto-regulation) changed the ABP-to-ICP transmission into a passive and linear pressure transmission [27-29]. Such disrupted cerebral pressure regulation would cause the ABP waves to correlate positively with the ICP waves. This is our rational for considering the MWA_ABP_/MWA_ICP _correlation as an indicator of the state of cerebrovascular pressure regulation. According to this concept, when the cerebrovascular pressure auto-regulation is disrupted, the correlation between ABP and ICP wave amplitudes becomes positive. The method of computing moving MWA_ABP_/MWA_ICP _correlations was first applied to patients with head injury [33] and iNPH [34].

#### The association between CO and ICP wave amplitudes

It is suggested that the association between CO and ICP wave amplitudes (CO/MWA_ICP _correlation) depends on the state of cerebrovascular pressure regulation (indicated by the MWA_ABP_/MWA_ICP _correlation). During disrupted cerebrovascular pressure auto-regulation, CO would directly affect the ICP wave amplitude levels (causing the CO/MWA_ICP _correlation to become positive and approach +1), as opposed to a situation of intact cerebrovascular pressure auto-regulation when CO would less likely affect the ICP wave amplitude levels (causing the CO/MWA_ICP _correlation to become non-significant or negative approaching -1). In line with this, in patients with a significant positive CO/MWA_ICP _correlation, the MWA_ABP_/MWA_ICP _correlation was also significantly positive (Table 4).

It should be noted that a clinically-significant shunt response was seen in patients with increased ICP wave amplitudes even though the recordings were accompanied by significant positive CO/MWA_ICP _correlations or significant positive MWA_ABP_/MWA_ICP _correlations. This latter observation compares with recent reports of no significant differences in MWA_ABP_/MWA_ICP _correlations between iNPH patients with clinical response to shunting (high ICP wave amplitudes) or no clinical response (low ICP wave amplitudes) [34]. This author finds it reasonable to speculate that when iNPH patients present with both impaired intracranial compliance (indicated by high ICP wave amplitudes) and impaired cerebrovascular pressure regulation (indicated by positive MWA_ABP_/MWA_ICP _correlation), the response to shunting is primarily determined by the improvement in intracranial compliance following CSF diversion.

In this cohort, a significant negative correlation between CO and ICP wave amplitude was seen in 16 of 29 patient recordings (55%); 13 of the 16 recordings were in group A (high ICP wave amplitudes) showing that in patients with high MWA_ICP_, a negative association between CO and MWA_ICP _predominates. Based on some information in the literature, it is tempting to speculate that such a negative correlation between CO and ICP wave amplitudes have relevance for the pathophysiology of iNPH. Following induction of experimental hydrocephalus in dogs, the ICP wave amplitudes increased (indicative of reduced intracranial compliance) and the CO became reduced, causing a negative correlation between CO and ICP wave amplitudes [20]. They also observed a global reduction in CBF, as well as reduction of blood flow in cardiac tissue and evidence of congestive heart failure [20]. They suggested that the reduced CBF seen in animals with experimental hydrocephalus was secondary to reduced CO. This is an interesting hypothesis given the many studies showing reduced global and/or regional CBF in iNPH [4-7]. Thus, if reduced CO reduces cerebral blood flow (CBF), it is possible that the reduced CBF seen in iNPH is at least partly heart-mediated. If so, iNPH can be considered a systemic disease involving cardiovascular alterations. It should be noted, however, that the past literature is somewhat inconsistent regarding the association between CO and CBF, and it depends on the state of the cerebral auto-regulation [48-51].

## Conclusions

While this overall dataset from iNPH patients gave no evidence of increased CO or ABP wave amplitude levels in patients with increased ICP wave amplitudes, analysis of recordings from individual patients provide evidence that an association between CO and ICP wave amplitudes is partly related to the association between ABP and ICP wave amplitudes (which can be indicative of the state of cerebrovascular pressure regulation), and partly related to the levels of ICP wave amplitude (which can be indicative of the intracranial compliance). In this cohort, shunt response was primarily related to level of ICP wave amplitudes. Further research is needed to clarify how CO associates with the intracranial pressure dynamics in hydrocephalus patients.

## List of abbreviations

ABP: Arterial blood pressure; CBF: Cerebral blood flow; CBV: Cerebral blood volume; CO: Cardiac output; CSF: Cerebrospinal fluid; DO_2_: oxygen consumption; ICP: Intracranial pressure; HR: Heart rate; ICC: Intracranial compliance; iNPH: Idiopathic normal pressure hydrocephalus; MWA_ABP_: Mean arterial blood pressure wave amplitude; MWA_ICP_: Mean ICP wave amplitude; SV: Stroke volume; SVR: systemic vascular resistance.

## Competing interests

The software used for analysis of the ICP recordings (Sensometrics Software) is manufactured by a software company (dPCom AS, Oslo) wherein Per Kristian Eide MD PhD has a financial interest.

## Authors' contributions

PKE: sole author. The author has read and approved the final version of the manuscript.

## Supplementary Material

Additional file 1 Table S1 The NPH Grading Scale used at the Department of Neurosurgery, Oslo University Hospital - Rikshospitalet Click here for file
