# Supplementary material for: Cardiac output in idiopathic normal pressure hydrocephalus: association with arterial blood pressure and intracranial pressure wave amplitudes and outcome of shunt surgery
Source: Fluids Barriers CNS. 2011 Feb 4;8:11. doi: 10.1186/2045-8118-8-11 (PMC3044095; doi:10.1186/2045-8118-8-11)
Supplement: Additional file 1 — Table S1 The NPH Grading Scale used at the Department of Neurosurgery, Oslo University Hospital - Rikshospitalet [file 2045-8118-8-11-S1.DOC]

**Table S1. The NPH Grading Scale used at the Department of Neurosurgery, Oslo University Hospital - Rikshospitalet**

| Scale | Clinical performance | NPH Score |
| --- | --- | --- |
| ***Gait disturbance*** | | |
| 5 | Normal gait. |  |
| 4 | Gait is abnormal, but walking is possible without support. Imbalance when turning with short steps. Widened base and occasional falling. |  |
| 3 | A cane is needed. Independent walking is possible but is unstable or the patient falls. |  |
| 2 | Support from another person is needed. Ambulating is possible with help. |  |
| 1 | Patient is bedridden or not able to ambulate. |  |
| ***Urinary incontinence*** | | |
| 5 | No subjective or objective incontinence. |  |
| 4 | Urinary urgency. Rare incontinence. |  |
| 3 | Occasional urinary incontinence. |  |
| 2 | Continuous urinary incontinence. |  |
| 1 | Both urinary and fecal incontinence. |  |
| ***Dementia*** | | |
| 5 | Normal. |  |
| 4 | Memory problems exist that are reported by patient or family. |  |
| 3 | Important memory problems with more or less severe behavior disturbances. |  |
| 2 | Severe dementia. |  |
| 1 | Vegetative. |  |
| 15 | Total score |  |
